# Flagellate Dermatitis: A Culinary Flogging

**DOI:** 10.5811/cpcem.2016.11.32785

**Published:** 2017-01-18

**Authors:** Samuel Y. Ko

**Affiliations:** Naval Hospital Camp Pendleton, Branch Heatlh Clinic, Group Aid Station, Yuma, Arizona

## CASE

A 30-year-old male, previously healthy, presented with a rash of three days duration. Lesions were first noticed on his right thigh, chest and back. The rash was intensely pruritic, but not painful. The patient tried self-treatment with oral diphenhydramine but had minimal relief. Review of systems was negative. He denied any sick contacts, recent travel, outdoor, chemical or other irritant exposures. Past medical history was noncontributory; the patient denied previous rashes. Exam was notable for a generalized rash in centripetal distribution. Lesions were raised, erythematous, and ranging in appearance from papules to wheals. Most striking was the crisscrossed linear pattern (Image 1a, 1b, 1c). The patient was initially treated for allergic reaction with topical hydrocortisone and oral hydroxyzine. He presented again two days later with worsening rash affecting knees, groin, and elbows, in a similar appearance. A detailed exposure history revealed consumption of multiple dishes containing Shiitake mushrooms 48 to 72 hours prior to onset at a restaurant serving Chinese cuisine. Furthermore, the patient had continued to eat leftovers after initial presentation.

Flagellate dermatitis, alternatively shiitake dermatitis, was diagnosed with positive history of ingestion of shiitake mushroom, *Lentinus edodes*. Shiitake is becoming more popular as a culinary ingredient not limited to Asian cuisine. Flagellate dermatitis is named for the flogged-like appearance, consistent with previous case reports.^1^ Close examination of the rash revealed a confluence of small papules into the linear streaks. Reports have been associated with ingestion of undercooked or raw mushroom.^1^ Pathophysiology remains poorly understood with evidence for both toxic reaction to lentinan polysaccharide and allergic-type hypersensitivity reaction.^2^ Biopsy and skin prick testing have been nonspecific and not validated for routine testing.^3^ Improvement was seen after similar treatment used in a previous case consisting of oral prednisone 50mg burst, oral cetirizine 10mg daily and topical mometasone furoate 0.1% daily.^4^ The rash resolved in approximately 10 days from start of treatment.

## Figures and Tables

**Image 1a:**
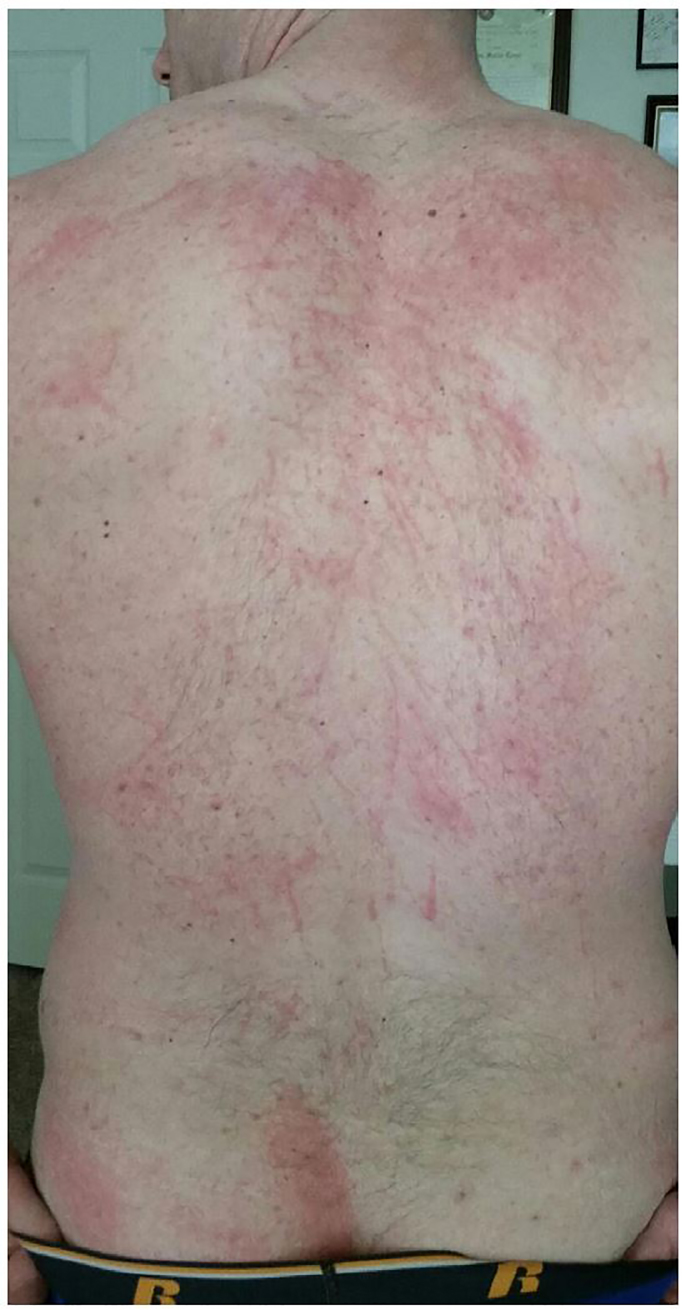
Shiitake dermatitis with characteristic flogged appearance.

**Image 1b:**
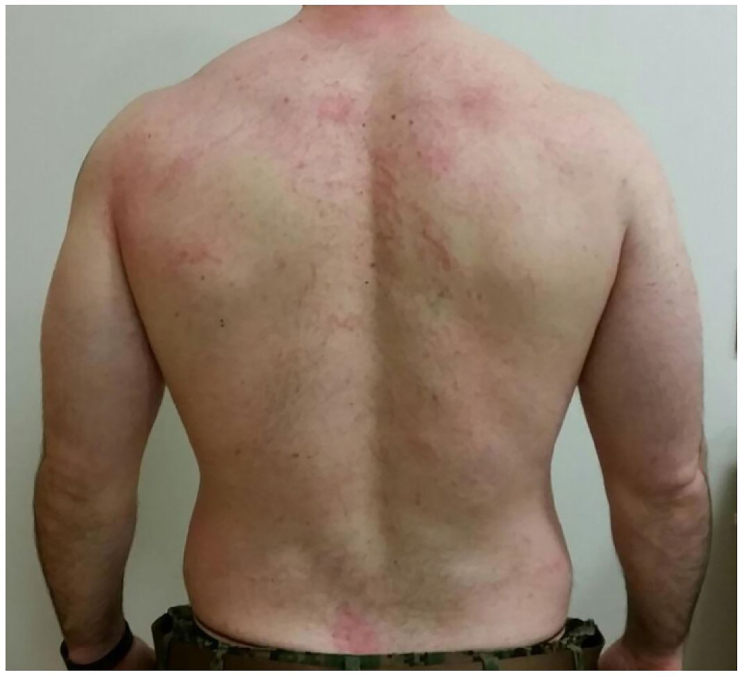
Sequential photo on day 2 after initiating steroid burst.

**Image 1c:**
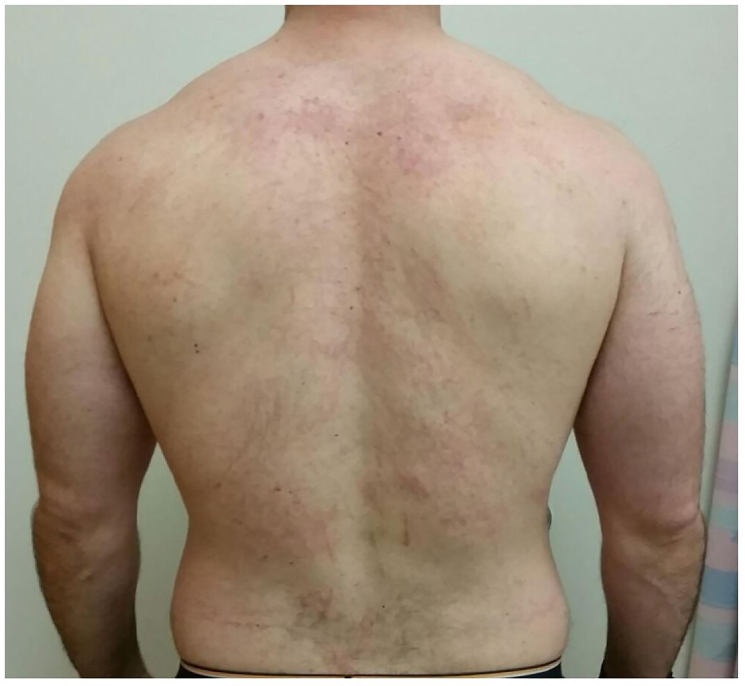
Sequential photo on day 5 after initiating steroid burst.
